# Facile Synthesis of Well-Dispersed Ni_2_P on N-Doped Nanomesh Carbon Matrix as a High-Efficiency Electrocatalyst for Alkaline Hydrogen Evolution Reaction

**DOI:** 10.3390/nano9071022

**Published:** 2019-07-17

**Authors:** Fan Yang, Shuo Huang, Bing Zhang, Liqiang Hou, Yi Ding, Weijie Bao, Chunming Xu, Wang Yang, Yongfeng Li

**Affiliations:** State Key Laboratory of Heavy Oil Processing, China University of Petroleum (Beijing), Beijing 102249, China

**Keywords:** N-doped carbon, Ni_2_P, hydrogen evolution reaction

## Abstract

The development of non-noble metal hydrogen evolution catalysts that can replace Pt is crucial for efficient hydrogen production. Herein, we develop a type of well-dispersed Ni_2_P on N-doped nanomesh carbon (NC) electrocatalyst by a facile pyrolysis method, which shows excellent hydrogen evolution reaction (HER) catalytic performance. It is rather remarkable that the overpotential of Ni_2_P/NC prepared under optimal proportion is 108 mV at 10 mA·cm^−2^ current density in 1 M KOH solution with the tafel slope of 67.3 mV·dec^−1^, the catalytic activity has no significant attenuation after 1000 cycles of cyclic voltammetry (CV)method. The hydrogen evolution performance of the electrocatalytic is better than most similar catalysts in alkaline media. The unique mesh structure of the carbon component in the catalyst facilitates the exposure of the active site and reduces the impedance, which improves the efficiency of electron transport as well as ensuring the stability of the hydrogen evolution reaction. In addition, we prove that nitrogen doping and pore structure are also important factors affecting catalytic activity by control experiments. Our results show that N-doped nanomesh carbon, as an efficient support, combined with Ni_2_P nanoparticles is of great significance for the development of efficient hydrogen evolution electrodes.

## 1. Introduction

For the past several years, great changes have taken place in the environment due to the burning of fossil fuels. As alternatives to fossil fuels, many new environment-friendly energy sources have been developed, such as water energy, wind energy, hydrogen energy, and so on [1]. Hydrogen energy, as a kind of energy with high energy density and no pollution, has attracted extensive attention [2,3,4]. It mainly comes from the following sources: coal gasification, natural gas reformation, organisms and electrolysis of water. Among them, hydrogen production by water electrolysis has the advantages of abundant raw materials, high purity and no emissions of polluting gas. Moreover, electrolysis of water to produce hydrogen is regarded as an effective way to solve the current environmental pollution and energy crises [5,6]. Electrolysis of water without catalyst needs to overcome a large energy barrier. In order to reduce the energy consumption in the hydrogen evolution reaction (HER) process and accelerate the rate of HER, platinum and other noble metal catalysts with low overpotential and Tafel slope have been developed [7,8,9]. However, the scarcity and high cost of precious metal based catalysts limit their commercial use. Therefore, the development of high-efficiency non-noble metal catalytic electrodes for hydrogen evolution is of great significance.

Transition metal compounds, such as carbides [10,11], sulfides [12], and phosphides [13,14], are important catalysts for the HER. Among them Ni_2_P has proton-acceptor and hydride-acceptor, combining the high thermostability of extended surfaces and the high catalytic activity of the hydrogenase. In addition, the P sites provide moderate bonding to the HER intermediates, thus enhancing the rate of the reaction [15].

In recent years, Ni_2_P with different structures has been widely studied and applied in hydrogen evolution reactions, for example, MOF-derived Ni_2_P [16], Ni_2_P nanosheets [17], self-supported Ni_2_P [18], which have been proven to show high hydrogen evolution catalytic activity. However, there is still a gap between Ni_2_P and noble metal catalysts. Researchers have made many attempts to improve the catalytic activity of Ni_2_P. It is widely known that the conductivity and specific surface area of materials are important factors to promote electrocatalytic efficiency. However, some catalysts developed at present have the disadvantages of poor conductivity and high impedance, so improving the conductivity of materials is still a problem to be solved. Consequently, some carbon materials become ideal supports for electrocatalytic reactions due to their high conductivity and specific surface area [3,19,20,21,22]. Moreover, the addition of certain carbon materials can protect the catalytic activity center and improve the catalytic stability [23,24,25,26,27]. Many researchers combine carbon materials with nickel phosphide for hydrogen evolution reactions, and these composites show excellent catalytic properties, stability, and electrical conductivity, such as Ni_2_P/NRGO (N-doped reduced graphene oxide) [28] and Ni_2_P/CNS (carbon nanosheets) [29]. It is of great research value to develop a high-efficiency carbon material as a support for HER.

The porous carbon materials that are synthesized by chemical vapor deposition (CVD) method using MgO-templated can be used for hydrogenation of nitroarenes [30], aerobic oxidative coupling of amines [31], and lithium-ion batteries [32] with high performance, due to their high specific surface area and abundant active sites. In addition, this type of carbon material can also be used as an efficient support with Pd/PdO nanoparticles attached for methanol oxidation [33]. However, composite materials produced by this method have never been used in catalysis of hydrogen evolution reactions. Herein, the N-doped nanomesh carbon with well-dispersed Ni_2_P (Ni_2_P/NC) is developed for the first time by a facile pyrolysis method with cost-effective NiCl_2_·6H_2_O and NaH_2_PO_2_ [34]. Different N-doped nanomesh carbon (NC) content, as a vital variable, is used to adjust the morphology of the material. Furthermore, we have made intensive studies of linear sweep voltammetry, electrochemical impedance spectroscopy, cyclic-voltammetry, and so on. The appropriate content of this N-doped material as the substrate can significantly reduce the hydrogen evolution overpotential of nickel phosphite and reduce the impedance in the reaction process, which is high-efficiency due to its huge specific surface area, abundant active sites and excellent electrical conductivity.

## 2. Materials and Methods

### 2.1. Materials

All reagents were used as received.

Ethanol (Sinopharm Chemical Reagent Beijing Co., Ltd., Beijing, China), Sodium Hypophosphite (NaH_2_PO_2_, 99%, Innochem Technology Co., Ltd., Beijing, China), Nickel chloride hexahydrate (NiCl_2_·6H_2_O, 99.9%, Sigma-Aldrich, Shanghai, China), Potassium hydroxide (KOH, Sinopharm Chemical Reagent Beijing Co., Ltd., Beijing, China), Light magnesium oxide (MgO, Sinopharm Chemical Reagent Beijing Co., Ltd., Beijing, China), 1,10-Phenanthroline (C_12_H_8_N_2_, J&K Scientific, Beijing, China), Nafion solution (Sigma-Aldrich, Shanghai, China), Triphenylmethane, (C_19_H_16_, J&K Scientific, Beijing, China), 20 wt % Pt on Vulcan XC-72R (Johnson Matthey Corporation, Beijing, China). 

### 2.2. Synthesis of NC

The MgO template was first synthesized according to a standard method [31]. Then 3 g 1, 10 -phenanthroline was dissolved in 100 mL ethanol and continuously stirred at room temperature and pressure for 30 min. Subsequently, 5 g template MgO was added into the solution and stirred overnight. After the solvent was removed by rotary evaporation, the mixture was annealed in Ar atmosphere and kept at 750 °C for 2 h with a 10 °C/min heating rate. Finally, the black powder was washed with 1 M diluted hydrochloric acid and then with deionized water several times until pH = 7.

### 2.3. Synthesis of Bulk Ni_2_P

First, 1 mmol NiCl_2_·6H_2_O and 1.5 mmol NaH_2_PO_2_ were dissolved in 25 mL deionized water, the mixture stirred for 4 h and dried under 90 °C for 12 h. The mixture was annealed in Ar atmosphere and kept at 300 °C for 30 min with a 5 °C/min heating rate. Finally, the powder was washed with deionized water and ethanol several times and dried under 120 °C for 3 h [31,34]. 

### 2.4. Synthesis of Ni_2_P/NC

First, 1 mmol NiCl_2_·6H_2_O and 1.5 mmol NaH_2_PO_2_ were dissolved in 25 mL deionized water. Then NC in different amounts (20 mg, 40 mg, 60 mg, 80 mg) were added, and the mixture was stirred for 4 h. Then the mixture was put in an ultrasonic bath for 60 min and freeze-dried for 18 h. The mixture was annealed in Ar atmosphere and kept at 300 °C for 30 min with a 5 °C/min heating rate. Finally, the powder was washed with deionized water and ethanol several times, and dried under 120 °C for 6 h. We define the names of materials according to the amount of NC added, which are Ni_2_P/NC-20, Ni_2_P/NC-40, Ni_2_P/NC-60 and Ni_2_P/NC-80, respectively. For comparison, Ni_2_P/C-60 and Ni_2_P/NG-60 catalysts were prepared by using triphenylmethane and NG [35] as carbon sources in the same way.

### 2.5. Characterizations

The obtained products are characterized by scanning electron microscopy (SEM, Gemini 300, ZEISS, Oberkochen, Germany), transmission electron microscopy (HRTEM, Tecnai G2 F20, FEI, Hillsboro, OR, USA) combined with an energy dispersive X-ray spectroscopy (EDS), X-ray diffraction (XRD, Bruker D8 Advance, and the data is collected on a Shimadzu XD-3A diffractometer using Cu Ka radiation, Bruker, Karlsruhe, Germany), X-ray photoelectron spectroscopy (XPS, Thermo Fisher K-Alpha American with an Al K X-ray source, Thermo Scientific, Waltham, MA, USA), and Raman spectroscopy (532 nm laser, Jobin Yvon T6400, Horiba Scientific, Kyoto, Japan).

### 2.6. Electrochemical Measurements

All electrochemical performance tests are performed in a classical glass cell (100 mL, Shanghai Yueci Electronic Technology Co., LTD, Shanghai, China) using the electrochemical workstation CHI 760E (Shanghai Chenhua Instrument Co., LTD, Shanghai, China). The device is composed of a standard three-electrode system: A glassy carbon electrode (diameter D = 3 mm) for the working electrode; a graphite column electrode for the counter electrode; and a Hg/HgO electrode for the reference electrode. The catalyst ink is prepared by dispersing 5 mg of catalyst into 1 mL of the mixed solvent containing water, ethanol, and Nafion solution with a volumetric ratio of 650:300:50. For the preparation of the catalytic electrodes, 5 μL of the catalyst ink is loaded onto a glassy carbon electrode. The GC electrodes are naturally dried at room temperature. All tests are performed in a 1 M KOH (pH = 14) solution. Before the test, we use high purity N_2_ to remove dissolved oxygen. The temperature of the testing process is 25 °C. The conversion formula of the potential between the reference electrode and the working electrode is RHE = E (Hg/HgO) + 0.098 V + 0.059 pH [36]. All the HER results are IR-corrected.

## 3. Results and Discussion

**Scheme 1:**
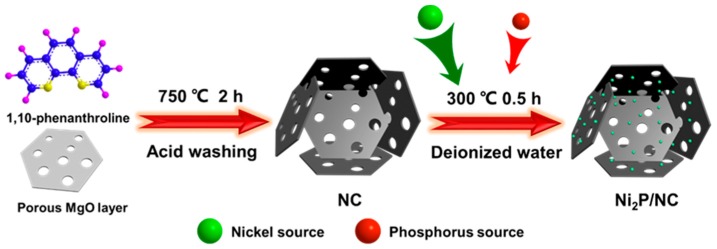
Illustration of the synthesis of Ni_2_P/NC.

The preparation process of Ni_2_P/NC is shown in Scheme 1. The NC is synthesized by typical MgO-templated chemical deposition (CVD) with phenanthroline as the nitrogen source and the carbon source. Then we add the nickel source and the phosphorus source, and the mixture is annealed in Ar atmosphere. Ni_2_P/NC is finally obtained after washing and drying.

**Figure 1:**
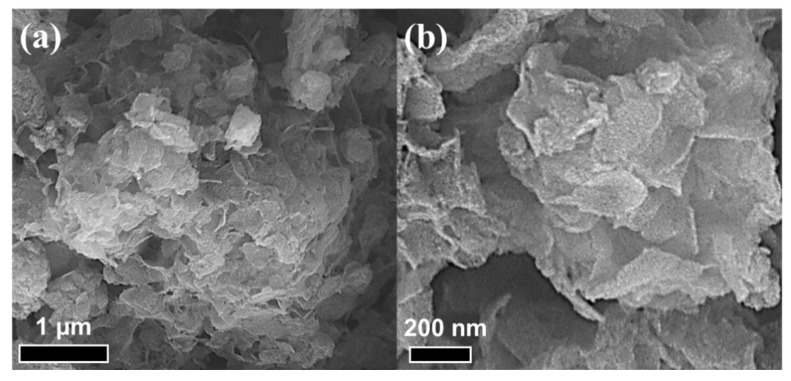
SEM images (**a**,**b**) of Ni_2_P/NC-60.

Firstly, NC is successfully synthesized by magnesium oxide template, its mesh structure is observed by SEM (Figure S1). Ni_2_P/NC-60 maintains a relatively complete lamellar appearance (Figure 1). It can be seen from Figure 1 that when the appropriate amount of NC is added, the load of Ni_2_P particles can be placed inside without damaging the original morphology of NC. Figures S1–S5 show the SEM images of a series of products. It is obvious that Ni_2_P is blocky when NC is not added. With the increase of NC addition, the morphology of the catalyst changed regularly, and the existing state of Ni_2_P on NC changed accordingly. Meanwhile, we use triphenylmethane and NG obtained by a typical hydrothermal method as the carbon source to prepare Ni_2_P/C and Ni_2_P/NG, for which the SEM images are shown in Figure S6a,b. It is obvious that the results showed that Ni_2_P particles are evenly distributed on the surface of C and NG.

**Figure 2:**
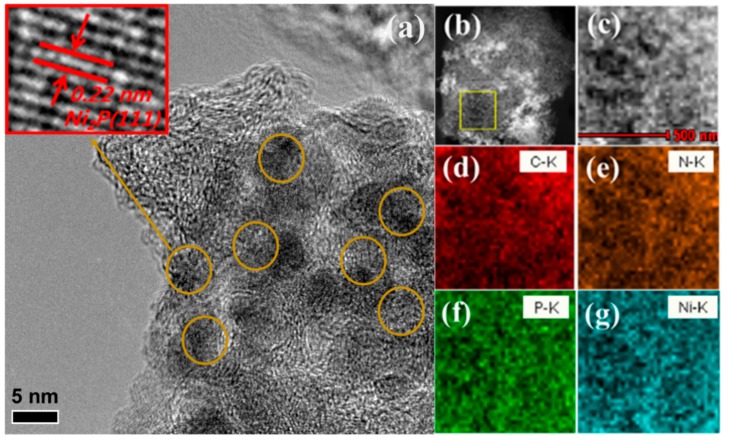
High resolution transmission electron microscopy (HRTEM) image (**a**) and energy dispersive X-ray spectroscopy (EDS) elemental mapping images (**b**–**g**) of Ni_2_P/NC-60.

The morphology and structure of Ni_2_P/NC-60 samples are further observed by high resolution transmission electron microscopy (HRTEM), as shown in Figure 2a. It can be seen that the addition of NC can disperse the Ni_2_P particles evenly, as there is no obvious aggregation of Ni_2_P particles. It is observed that the spacing between the lattice fringes is 0.22 nm, corresponding to the (111) crystal surface of Ni_2_P [37]. The energy dispersive X-ray (EDX) elemental mapping images (Figure 2b–g) further demonstrate that C, N, P and Ni elements are evenly distributed in the entire NC layer.

**Figure 3:**
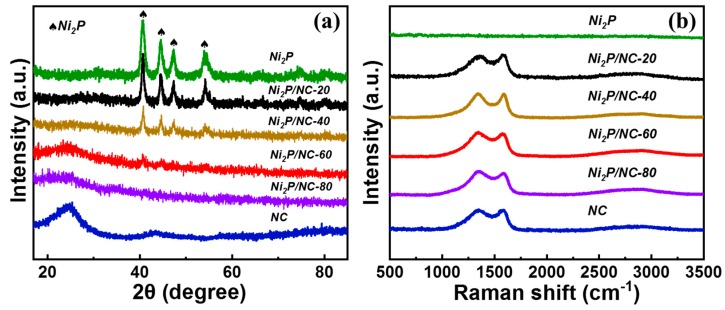
XRD patterns (**a**) and Raman spectra (**b**) of different components of materials.

In order to characterize the crystal structure, X-ray diffraction (XRD) tests are carried out on the materials (Figure 3a). The main diffraction peaks at 40.8°, 44.6°, 47.3°, 54.2° are matching well with the standard card of Ni_2_P (PDF 03-0953). It has been proven that Ni_2_P crystals can be successfully synthesized under experimental conditions [38]. In addition, a broad peak at 23.6° is also observed, which proves the formation of NC [31]. The intensity of Ni_2_P diffraction peaks in the figure decreases with the gradual increase of NC content, showing an obvious change trend. We speculate that the lower diffraction peaks are due to the small size and low content of Ni_2_P particles [34].

In addition, Raman spectroscopy (Figure 3b) is used to characterize the samples. There are two very distinctive D and G bands at about 1300 and 1600 cm^−1^, respectively. The D band arises from structural defects in the graphitic plane, whereas the G band is related to the E_2g_ vibrational mode of the sp^2^-bonded graphitic carbons [39]. The strength ratio I_D_/I_G_ of the D peak and the G peak indicates the defect degree of the material, the ratio of the Ni_2_P/NC material is all around 1.00, proving that the prepared material has abundant defect sites to provide sufficient reaction sites [40]. In addition, the weak and broad peaks at around 2700 cm^−1^ can be attributed to the combination of 2D band and D + G band of the NC [41]. Pure Ni_2_P has no obvious Raman peak, which is consistent with the results reported previously [42,43], which presumably has something to do with its particular molecular symmetry.

N_2_ adsorption-desorption measurement is used to further study the surface physical structures. The Brunauer-Emmett-Teller (BET) specific surface area (SSA) of pure Ni_2_P is 2.4 m^2^·g^−1^, indicating that the particle accumulation is relatively dense and there is almost no pore structure in the material. However, the BET SSA of the NC is 1340 m^2^·g^−1^ (Figure S7a,b). This result proves that the NC has large specific surface area and a porous structure, which is conducive to uniform dispersion of Ni_2_P particles. Besides, the BET SSA of the Ni_2_P/NC-60 is 633.5 m^2^·g^−1^, indicating that Ni_2_P is dispersed in the NC and occupies some of the pore structure [44]. The average pore radius is about 3 nm (Figure S8c), confirming the nanoporous structure of the Ni_2_P/NC [45].

**Figure 4:**
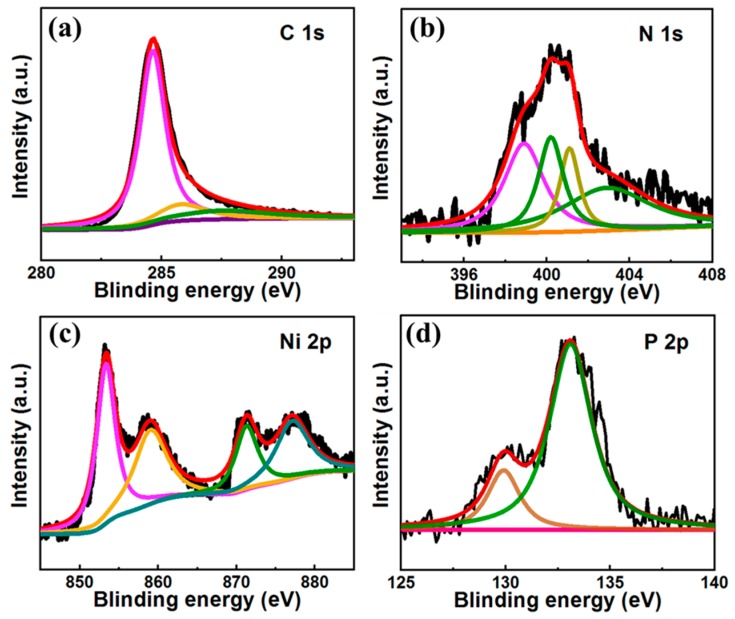
X-ray photoelectron spectroscopy (XPS) spectra of (**a**) C, (**b**) N, (**c**) Ni and (**d**) P.

In order to characterize the chemical state of the catalyst surface, X-ray photoelectron spectroscopy (XPS) measurements were carried out, the results for which are shown in Figure 4a–d. Figure S8 revealed the presence of the elements C, N, P, Ni and O, which further indicates the formation of Ni_2_P/NC-60.

By using a Gaussian fitting method, the C 1s spectrum (Figure 4a) shows three peaks at 284.6, 285.8 and 287.5, which can correspond to graphite-like sp^2^C, N-sp^2^C and N-sp^3^C of the NC [46,47]. As for the N 1s spectra (Figure 4b), they are decomposed with four peaks. The binding energy at 398.9 eV, 400.2 eV, 401.1, 402.9 can be consistent with pyridinic N, pyrrolic N, graphitic N and oxidized N in the catalyst [28,48]. The peak centered at 853.3 eV accompanied by a satellite peak at 859.0 eV corresponds to Ni 2p 3/2, and the peak at 871.3 eV together with a satellite peak at 877.1 eV is in line with Ni 2p 1/2 (Figure 4c), which are in good consistence with the characteristic peaks of Ni signals in Ni_2_P [17,28,49]. Besides, the binding energy of P 2p at 129.40 eV in Figure 4d is typical of metal-P bonds (i.e., Ni_2_P) [38]. The binding energy at 133.10 eV can be attributed to the phosphorus with a higher oxide state of phosphate at the surface [49,50,51]. In addition, XPS results also reveal that the mass ratio of Ni_2_P is about 21% in Ni_2_P/NC-60 (Table S1).

**Figure 5:**
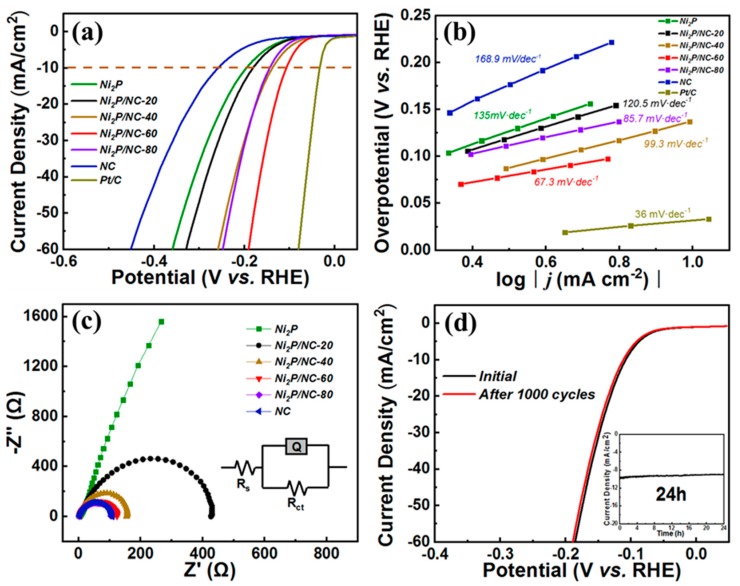
Profiles of different electrocatalytic experiments: (**a**) Linear sweep voltammetry (LSV) curves of Pt/C, Ni_2_P, Ni_2_P/NC-20, Ni_2_P/NC-40, Ni_2_P/NC-60, Ni_2_P/NC-80 and NC electrodes in 1 M KOH solution. (**b**) Tafel plots of Pt/C, Ni_2_P, Ni_2_P/NC-20, Ni_2_P/NC-40, Ni_2_P/NC-60, Ni_2_P/NC-80 and NC. (**c**) Nyquist plots of the Ni_2_P, Ni_2_P/NC-20, Ni_2_P/NC-40, Ni_2_P/NC-60, Ni_2_P/NC-80 and NC electrodes in 1 M KOH with a fitted equivalent circuit (inset). (**d**) LSV curves of Ni_2_P/NC-60 before and after 1000 cycles, the inset is the time dependence of current density curve at the potential of −105 mV vs. RHE in 1 M KOH.

Furthermore, we used linear sweep voltammetry (LSV) to test the hydrogen evolution performance of the composite in 1 M KOH solution [14,36,40]. Figure 5a shows the polarization curves of Pt/C, Ni_2_P, Ni_2_P/NC-20, Ni_2_P/NC-40, Ni_2_P/NC-60, Ni_2_P/NC-80 and NC in 1 M KOH solution. Obviously, the commercial Pt/C (20 wt %) catalyst shows superior catalytic activity, as the initial potential of hydrogen evolution only needs to reach 20 mV. The catalytic activity of pure Ni_2_P has poor performance, the overpotential is 210 mV at the current density of 10 mA·cm^−2^, which is basically consistent with the previously reported measurement values [52]. With the addition of NC, the catalytic activity first increased and then decreased. Under the current density of 10 mA·cm^−2^, the overpotential of Ni_2_P/NC-20, Ni_2_P/NC-40 and Ni_2_P/NC-60 are 180 mV, 137 mV and 108 mV, respectively. Thus, we can see that Ni_2_P/NC-60 has the best catalytic activity among them. The catalytic performance of the material is better than that of most of the previously reported Ni_2_P combined with carbon substrates catalysts for hydrogen evolution reaction (Table S2). Due to the different electronegativity of C and N, nitrogen doping can change the electronic state of carbon material structure, providing a resistance-free way for electrons to pass quickly through the layers of carbon material [48,53]. NC and Ni_2_P particles form a synergistic effect, which is conducive to accelerating the catalytic reaction kinetics of the HER on its surface. However, when the additive of NC reaches 80 mg, the catalytic activity begins to deteriorate; the overpotential is 143 mV at the current density of 10 mA·cm^−2^ due to the reduced amounts of Ni_2_P. For comparison, we also conducted a series of control experiments. The overpotential of pure NC at 10 mA·cm^−2^ current density is 260 mV, proving that NC is a kind of material with excellent catalytic performance. As mentioned above, we have prepared Ni_2_P/C and Ni_2_P/NG. At the current density of 10 mA·cm^−2^, the catalytic performance of both samples is poorer than that of Ni_2_P/NC-60 (Figure S9). It indicates that N element doping is an important factor affecting catalytic activity, and the unique porous nanomesh structure is conducive to full contact between materials and solutions, thus effectively promoting the kinetics of the hydrogen evolution reaction.

Tafel slope is also an important parameter to study the catalytic properties of the hydrogen evolution reaction. According to electrode kinetics, the relation between hydrogen evolution overpotential and reaction current density is proposed by the Tafel formula [54]:

η = a + b log |j|,(1)

Figure 5b shows the Tafel slopes measured and calculated by Pt/C (20 wt %), Ni_2_P, Ni_2_P/NC-20, Ni_2_P/NC-40, Ni_2_P/NC-60, Ni_2_P/NC-80 and NC under the same condition of tests, they are 36 mV·dec^−1^, 120.5 mV·dec^−1^, 99.3 mV·dec^−1^, 67.3 mV·dec^−1^, 85.7 mV·dec^−1^ and 168.9 mV·dec^−1^, respectively. Ni_2_P/NC-60 has the lowest slope of Tafel curve except Pt/C, proving that Ni_2_P/NC-60 has higher catalytic activity and higher reaction rate, which corresponds to the Volmer-Heyrovsky mechanism [55,56,57].

Meanwhile, electrochemical impedance spectroscopy (EIS) is considered as an important tool for studying electrode kinetics in catalytic processes (Figure 5c) [58]. The illustration in Figure 5c shows a fitted simplified equivalent circuit diagram, in which R_ct_ represents the alternating impedance of the electrolyte interface. With the increase of NC content, the semicircle radius presented a gradually decreasing trend, indicating that the addition of NC could lead to higher electron transfer rate and faster catalytic kinetics of the catalyst [59].

Stability is another important criterion for evaluating the catalytic performance of electrodes [60]. Figure 5d is the LSV scanning diagram of Ni_2_P/NC-60 before and after the cyclic-voltammetric (CV) measurement with 1000 cycles in 1 M KOH solution. As can be seen from the figure, after 1000 cycles the overpotential of Ni_2_P/NC-60 at the current density of 10 mA·cm^−2^ increases by only 3 mV, indicating that Ni_2_P/NC-60 electrode has excellent electrochemical stability in the process of the hydrogen evolution reaction in alkaline solution. The illustration is the time dependence of the current density curve at constant potential. After 24 h of testing, the current density does not decrease significantly. The results show that the activity of the catalyst is stable under the constant voltage test [60].

**Figure 6:**
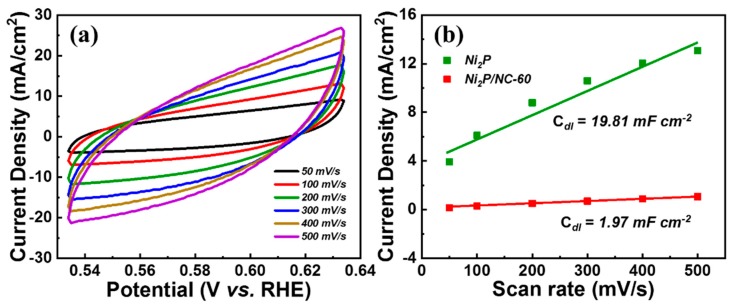
(**a**) CV curves for C_dl_ at different scan rates (50–500 mV s^−1^) of Ni_2_P/NC-60 in 1 M KOH; (**b**) Electrochemical double layer capacitance (C_dl_) at 0.584 V vs. RHE of Ni_2_P and Ni_2_P/NC-60.

In order to study the catalytic activity of Ni_2_P/NC-60 in the alkaline solution of the HER, we carried out CV scanning tests of pure Ni_2_P (Figure S10) and Ni_2_P/NC-60 (Figure 6a) at different scanning rates [14]. As shown in the figures, each curve presents a regular rectangle without obvious Faraday reaction. The result of electrochemical double layer capacitance (C_dl_) is shown in Figure 6b. On the basis of the calculation, the C_dl_ value of Ni_2_P/NC-60 is 19.81 mF·cm^−2^, while that of pure Ni_2_P is 1.97 mF·cm^−2^. The large ECSA value indicates that Ni_2_P/NC-60 has a large electrochemical active surface area, which is another reason for the high catalytic activity [14,42].

## 4. Conclusions

In conclusion, a series of novel Ni_2_P/NC catalysts were successfully designed and prepared by a facile pyrolysis method. Electrochemical tests show that by adding NC, hydrogen evolution overpotential, Tafel slope and AC impedance can be significantly reduced, the stability of the catalyst can be improved, the surface area of electrochemical activity can be increased. Among them, Ni_2_P/NC-60 has the most excellent hydrogen evolution performance, which is attributed to the fact that the addition of NC changes the original blocky morphology of the catalyst, prevents the aggregation of Ni_2_P particles, and exposes more active sites. In addition, the comparison experiment also proved that the porous structure of carbon material and the doping of N element are also important factors to improve its catalytic activity. Therefore, this work provides an effective method to prepare similar carbon-based non-noble metal hydrogen evolution catalysts.

## Supplementary Materials

The following are available online at https://www.mdpi.com/2079-4991/9/7/1022/s1, Figure S1: SEM image of NC, Figure S2: SEM image of Ni_2_P, Figure S3: SEM image of Ni_2_P/NC-20, Figure S4: SEM image of Ni_2_P/NC-40, Figure S5: SEM image of Ni_2_P/NC-80, Figure S6: SEM images of (a) Ni_2_P/NG and (b) Ni_2_P/C, Figure S7: (a) Nitrogen sorption isotherms. (b) SSA. (c) Pore diameters distributions, Figure S8: XPS spectra of Ni_2_P/NC-60, Figure S9: LSV of Ni_2_P/NC-60, Ni_2_P/NG and Ni_2_P/C in 1 M KOH, Figure S10: CV scanning of Ni_2_P in 1 M KOH at 50–500 mV s^−1^, Table S1: XPS Elemental analysis of Ni_2_P/NC-60, Table S2: Comparison of catalytic performance of different HER electrocatalysts in 1 M KOH.
