# Supplementary material for: Facile Synthesis of Well-Dispersed Ni2P on N-Doped Nanomesh Carbon Matrix as a High-Efficiency Electrocatalyst for Alkaline Hydrogen Evolution Reaction
Source: Nanomaterials (Basel). 2019 Jul 17;9(7):1022. doi: 10.3390/nano9071022 (PMC6669547; doi:10.3390/nano9071022)
Supplement: Supplementary file 1 [file nanomaterials-09-01022-s001.pdf]

Supplementary Information

Facile Synthesis of Well-Dispersed Ni_2P on N-Doped Nanomesh Carbon Matrix as a High-Efficiency Electrocatalyst for Alkaline Hydrogen Evolution Reaction

Fan Yang †, Shuo Huang †, Bing Zhang, Liqiang Hou, Yi Ding, Weijie Bao, Chunming Xu, Wang Yang and Yongfeng Li *

State Key Laboratory of Heavy Oil Processing, China University of Petroleum (Beijing), Beijing 102249, China

* Correspondence: yfli@cup.edu.cn; Tel.: +86-10-8973-3477

† These authors contributed equally to this work.

Figure S1.

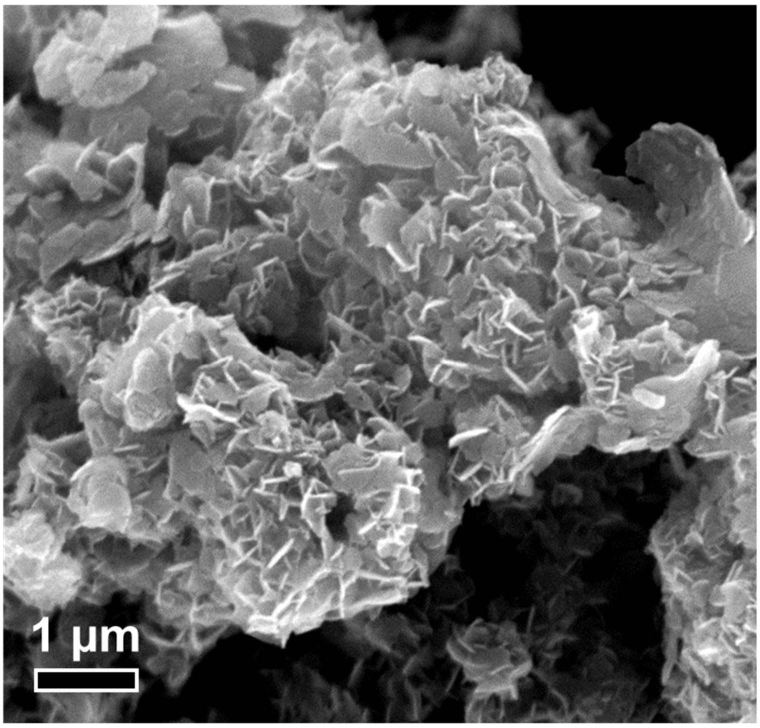

Figure S1. SEM image of NC.

Figure S2.

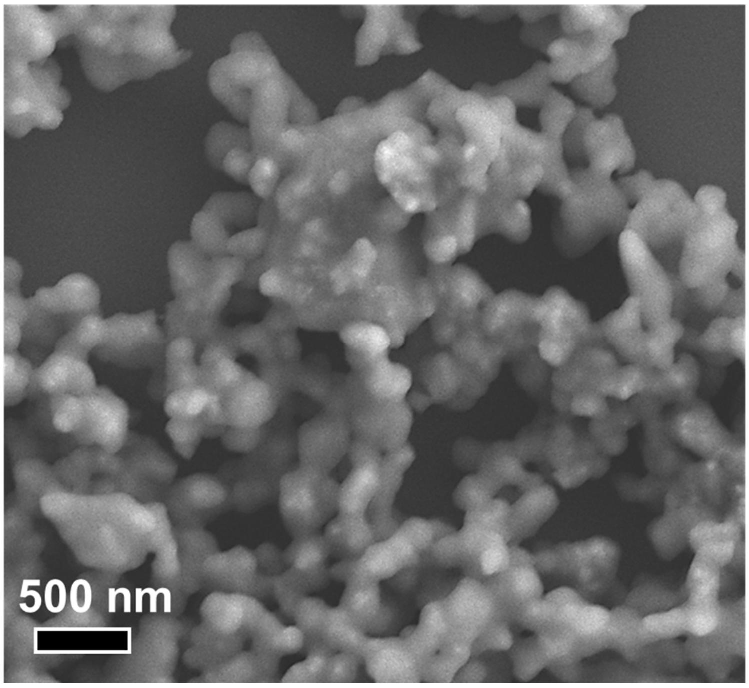

Figure S2. SEM image of Ni_2P.

Figure S3.

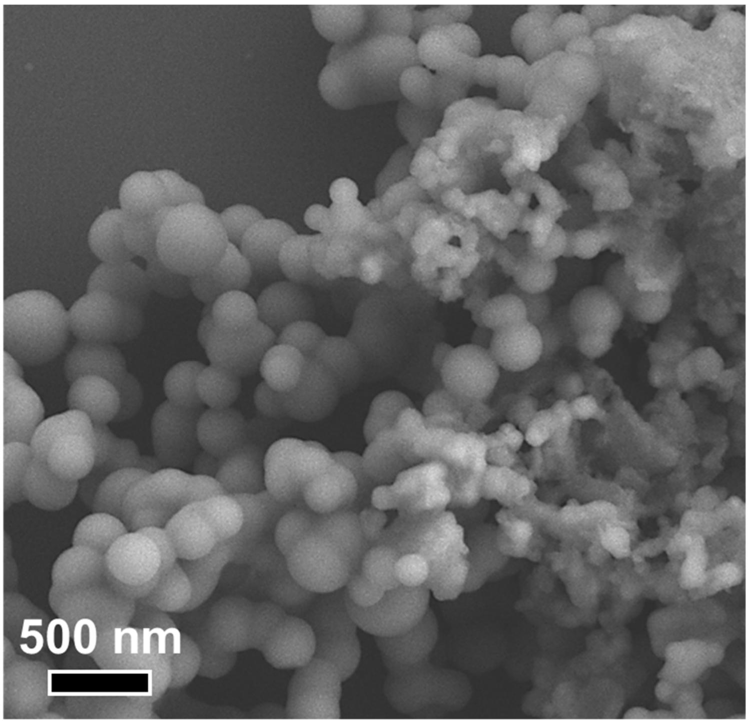

Figure S3. SEM image of Ni_2P/NC-20.

Figure S4.

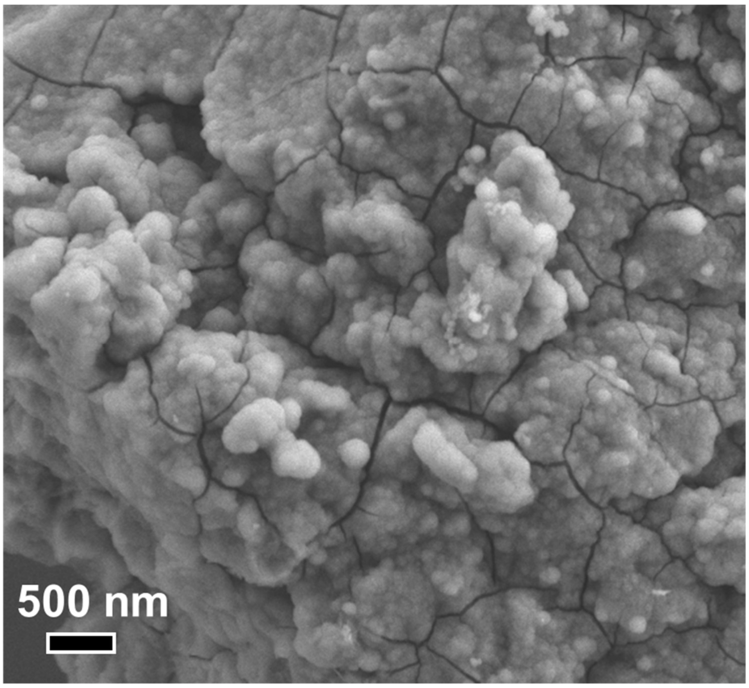

Figure S4. SEM image of Ni_2P/NC-40.

Figure S5.

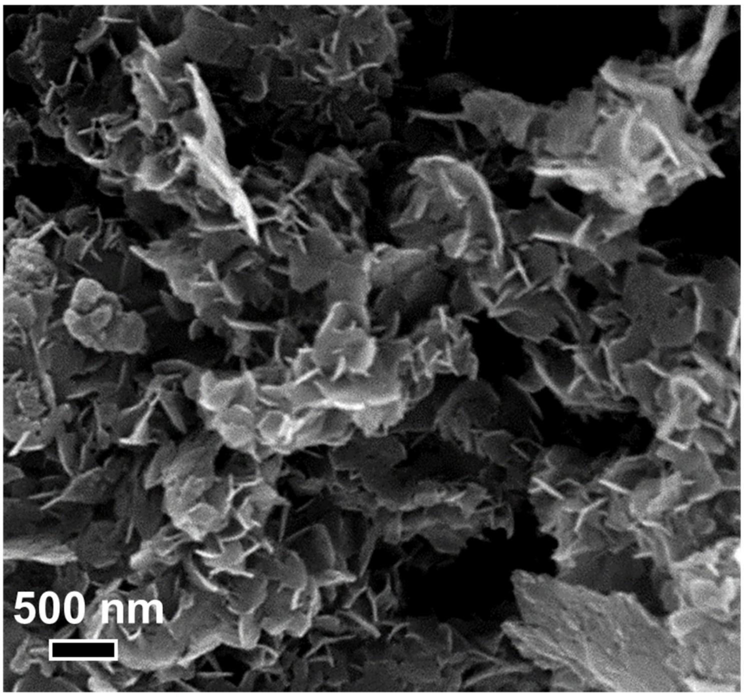

Figure S5. SEM image of Ni_2P/NC-80.

Figure S6.

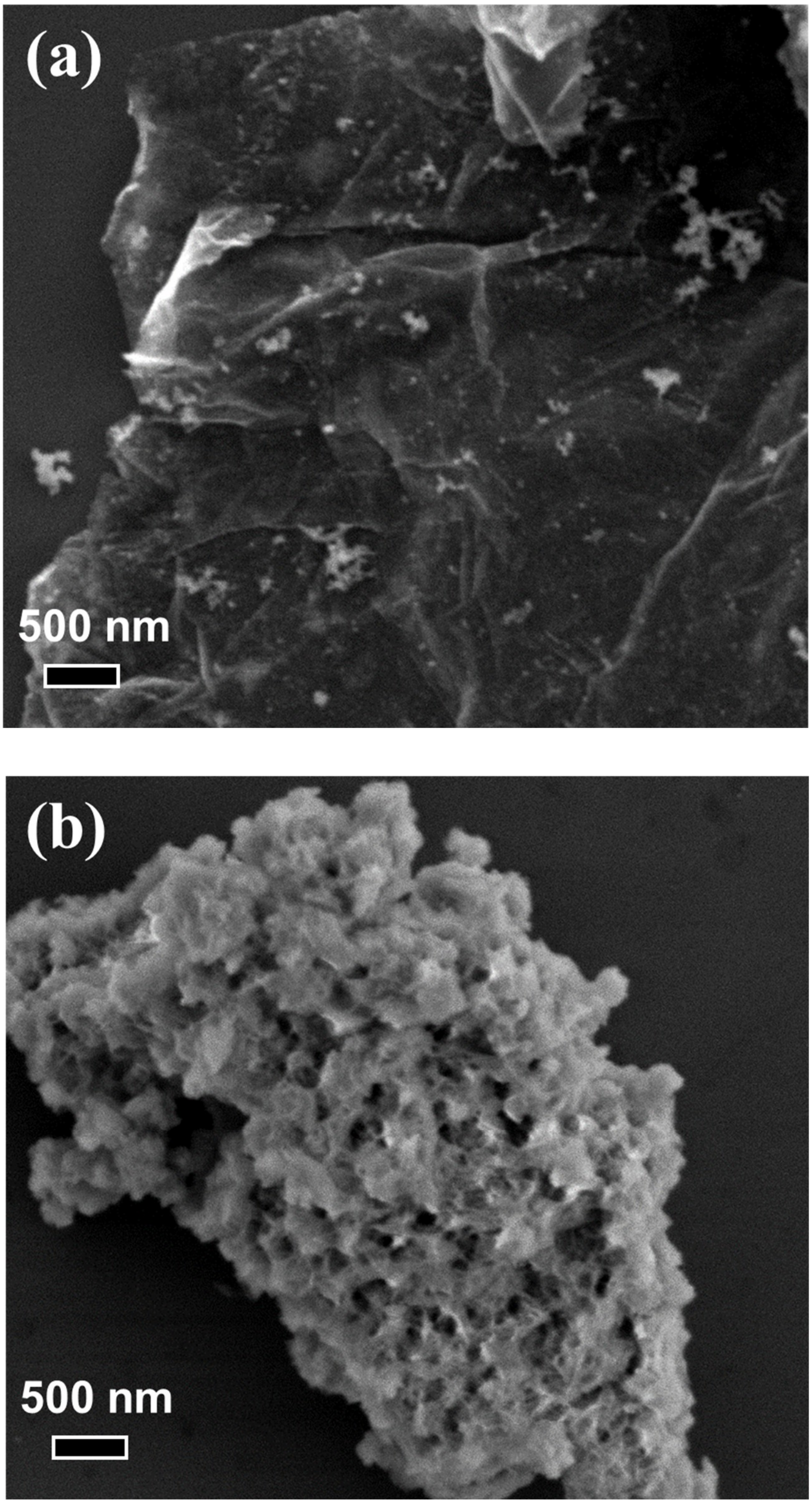

Figure S6. SEM images of (a)Ni_2P/NG-60 and (b)Ni_2P/C-60.

Figure S7.

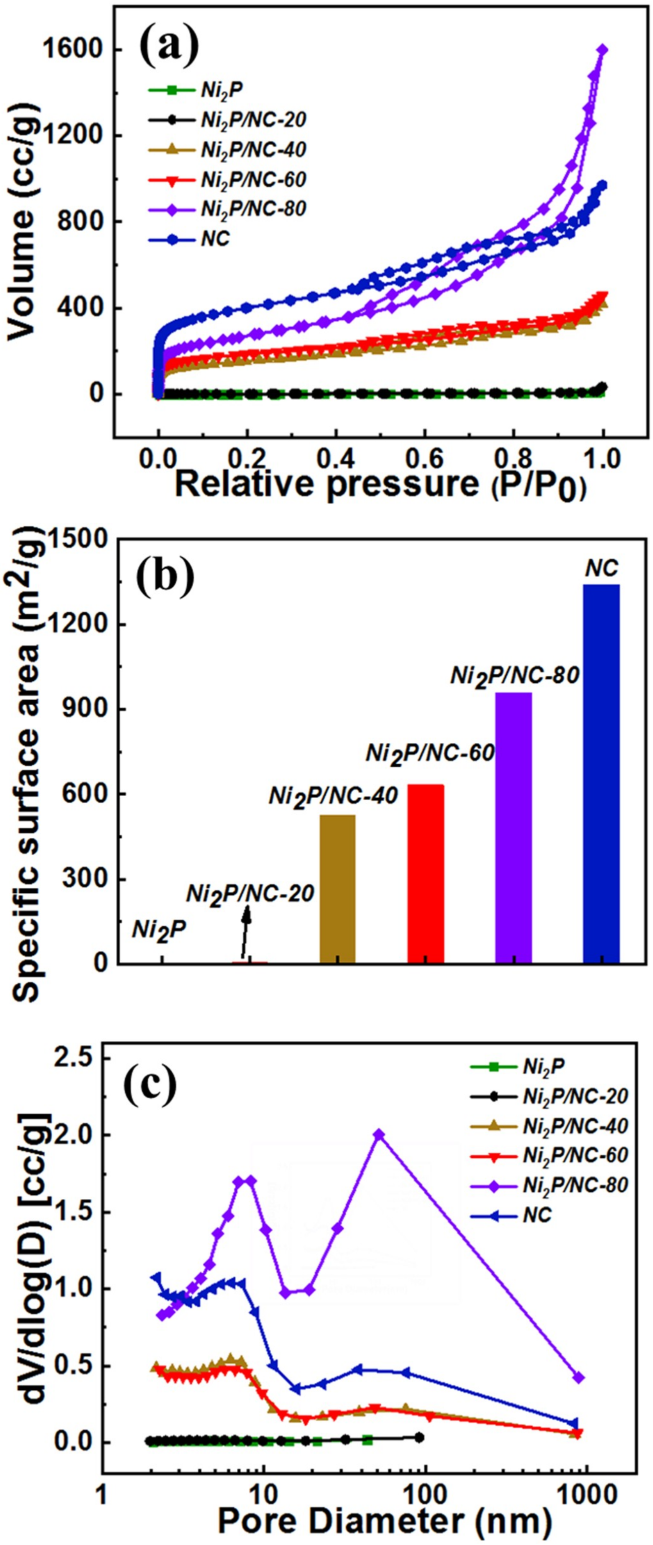

Figure S7. (a) Nitrogen sorption isotherms. (b) SSA. (c) Pore diameters distributions.

Figure S8.

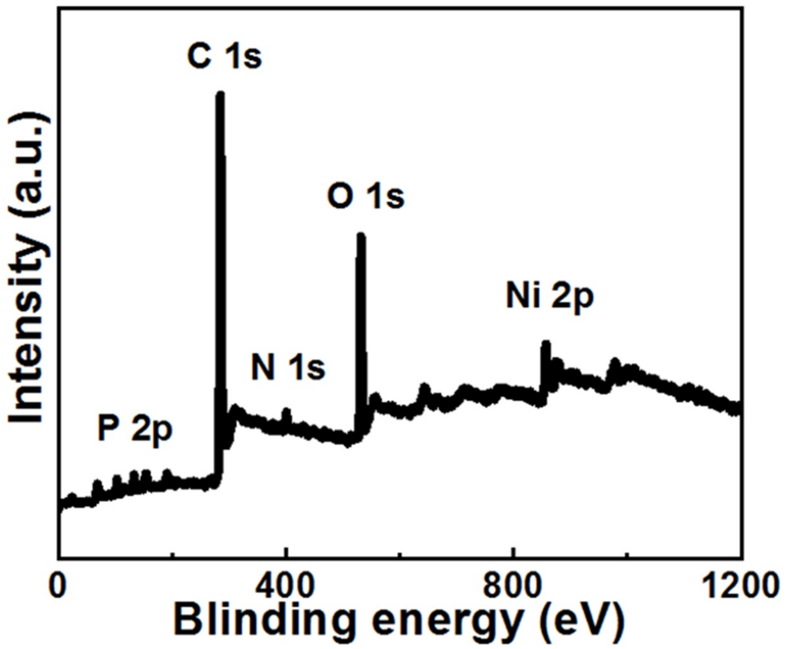

Figure S8. XPS spectra of Ni_2P/NC-60.

Table S1.

Table S1. XPS Elemental analysis of Ni_2P/NC-60.

Name	Atomic %	Weight %
C 1s	64.28	49.91
N 1s	6.06	5.49
Ni 2p	4.05	15.38
O 1s	22.79	23.59
P 2p	2.81	5.63

Table S2.

Table S2. Comparison of catalytic performance of different HER electrocatalysts in 1 M KOH.

Electrocatalyst	η (mV)	Current density (mA cm $^{-2}$)	Tafel slope (mV dec $^{-1}$)	Reference
This work	108	10	67.3	-
PrGO/NiCoP	106	10	58.3	[1]
G/MS-CS NTs	198	10	79	[2]
Ni_2P/Co_2P@NC	226	10	64.9	[3]
CoP@3D-NPC	203	10	71	[4]
Ni-GCS-2	205	10	111	[5]
CoP_2@3D-NPC-1.5	206	20	59	[6]
Ni/NCA-60	170 ± 2	10	74 ± 2	[7]
CoP/NiCoP/NC	75	10	64	[8]
Ra–Ni from Al and Ti	160	200(30℃)	-	[9]

1. Dong T.; Zhang X.; Wang P.; Chen H.S.; Yang P. Hierarchical nickel-cobalt phosphide hollow spheres embedded in P-doped reduced graphene oxide towards superior electrochemistry activity, *Carbon* 149 **2019**, 149, 222-233.
2. Wang X.; Zheng B.; Wang B.; Wang H.; Sun B.; He J.; Zhang W.; Chen Y. Hierarchical $MoSe_2$-$CoSe_2$ nanotubes anchored on graphene nanosheets: A highly efficient and stable electrocatalyst for hydrogen evolution in alkaline medium, *Electrochim. Acta* **2019**, 299, 197-205.
3. Zhang X.Y.; Guo B.Y.; Chen Q.W.; Dong B.; Zhang J.Q.; Qin J.F.; Xie J.Y.; Yang M.; Wang L.; Chai Y.M.; Liu C.G. Ultrafine and highly-dispersed bimetal Ni_2P/Co_2P encapsulated by hollow N-doped carbon nanospheres for efficient hydrogen evolution, *Int. J. Hydrogen Energy* **2019**, 44, 14908-14917.
4. Yang S.; Chen L.; Wei W.; Lv X.; Xie J. CoP nanoparticles encapsulated in three-dimensional N-doped porous carbon for efficient hydrogen evolution reaction in a broad pH range, *Appl. Surf. Sci.* **2019**, 476, 749-756.
5. Xu Y.; Ullah N.; Chen L.; Wei W.; Oluigbo C.J.; Xie M.; Zhang M.; Xie J. Nickel loaded graphene-like carbon sheets an improved electrocatalyst for hydrogen evolution reaction, *Mater. Chem. Phys.* **2019**, 227, 105-110.
6. Yang S.; Xie M.; Chen L.; Wei W.; Lv X.; Xu Y.; Ullah N.; Judith O.C.; Adegbemiga Y.B.; Xie J. Cobalt phosphide nanoparticles embedded in 3D N-doped porous carbon for efficient hydrogen and oxygen evolution reactions, *Int. J. Hydrogen Energy* **2019**, 44, 4543-4552.

7. Shanmugam P.; Murthy A.P.; Theerthagiri J.; Wei W.; Madhavan J.; Kim H.S.; Maiyalagan T.; Xie J. Robust bifunctional catalytic activities of N-doped carbon aerogel-nickel composites for electrocatalytic hydrogen evolution and hydrogenation of nitrocompounds, *Int. J. Hydrogen Energy* **2019**, 44, 13334-13344.
8. Boppella R.; Tan J.; Yang W.; Moon J. Homologous CoP/NiCoP Heterostructure on N-Doped Carbon for Highly Efficient and pH-Universal Hydrogen Evolution Electrocatalysis, *Adv. Funct. Mater.* **2018**, 29.
9. Colli A.N.; Girault H.H.; Battistel A. Girault. Non-Precious Electrodes for Practical Alkaline Water Electrolysis, Materials 2019, 12, 1336.

Figure S9.

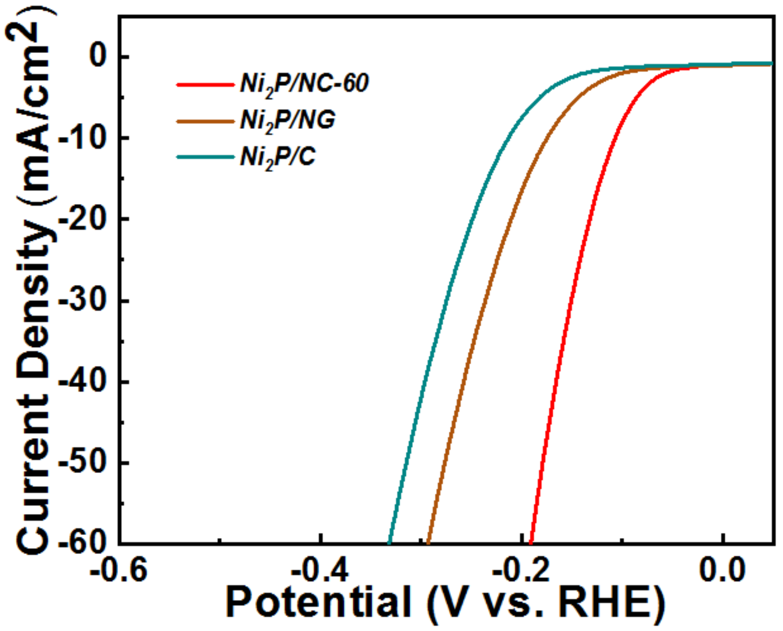

Figure S9. LSV of Ni_2P/NC-60, Ni_2P/NG and Ni_2P/C in 1 M KOH.

Figure S10.

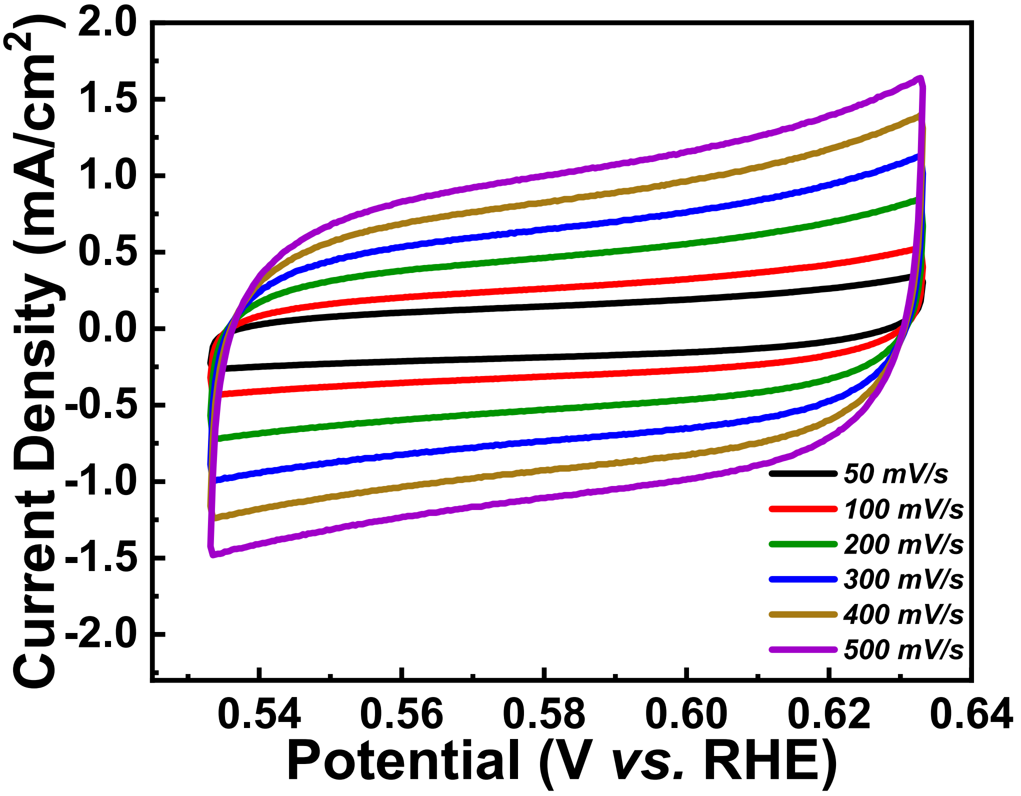

Figure S10. CV scanning of Ni_2P in 1 M KOH at 50-500 mV s^{-1}.
